# Genomic epidemiology of penicillin-non-susceptible *Streptococcus pneumoniae*

**DOI:** 10.1099/mgen.0.000305

**Published:** 2019-10-14

**Authors:** Tamsin C. M. Dewé, Joshua C. D’Aeth, Nicholas J. Croucher

**Affiliations:** ^1^​ MRC Centre for Global Infectious Disease Analysis, Department of Infectious Disease Epidemiology, St. Mary’s Campus, Imperial College London, London, W2 1PG, UK

**Keywords:** antibiotic resistance, pneumococcus, genomic epidemiology, penicillin

## Abstract

Penicillin-non-susceptible *Streptococcus pneumoniae* (PNSP) were first detected in the 1960s, and are now common worldwide, predominantly through the international spread of a limited number of strains. Extant PNSP are characterized by mosaic *pbp2x*, *pbp2b* and *pbp1a* genes generated by interspecies recombinations, with the extent of these alterations determining the range and concentrations of β-lactams to which the genotype is non-susceptible. The complexity of the genetics underlying these phenotypes has been the subject of both molecular microbiology and genome-wide association and epistasis analyses. Such studies can aid our understanding of PNSP evolution and help improve the already highly-performing bioinformatic methods capable of identifying PNSP from genomic surveillance data.

## Data Summary

The prevalences of penicillin-non-susceptible pneumococci across European countries were extracted from the European Centre for Disease Prevention and Control’s (ECDC) annual reports on Antimicrobial Resistance Surveillance in Europe. The data on consumption of penicillins across European countries were extracted from the ECDC’s annual reports on Surveillance of Antimicrobial Consumption in Europe. The editions used are specified in the Data bibliography.

**Impact Statement**

*
Streptococcus pneumoniae *, or the pneumococcus, causes a variety of diseases that have been routinely treated with penicillins since the 1940s. Penicillin-non-susceptible pneumococci (PNSP) were first detected in the 1960s, and multiple PNSP strains have since spread worldwide. PNSP continue to co-circulate with penicillin-susceptible pneumococci, despite changes in prescribing practices and vaccine introductions hoped to reduce the prevalence of resistant bacteria. This review highlights the role of genomics in improving our understanding of how PNSP evolved, enhancing our ability to trace their transmission, and developing new methods of predicting how pneumococci will respond to treatment with different penicillins.

## The burden of penicillin-non-susceptible pneumococcal disease

**Fig. 1.**
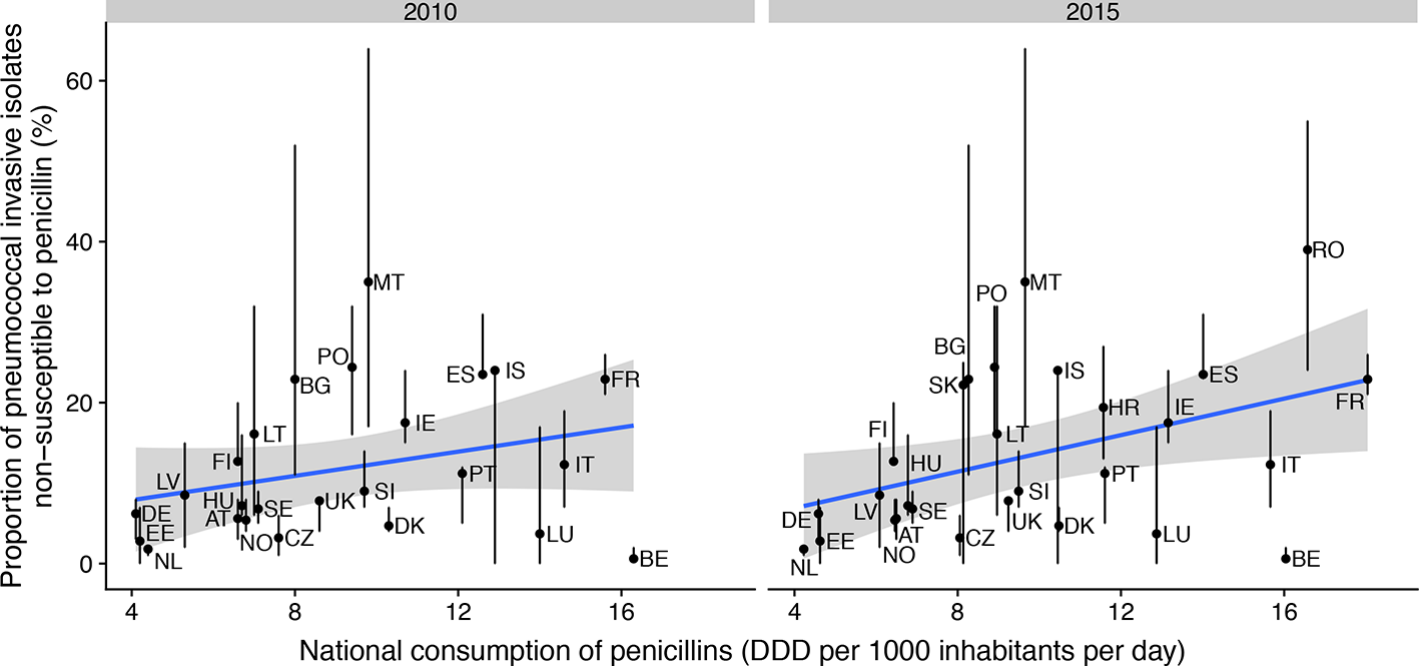
Correlation between outpatient consumption of penicillins and the proportion of invasive pneumococcal disease isolates that were non-susceptible to penicillin across European countries in 2010, when the 13-valent PCV superseded the 7-valent formulation in many countries, and 2015. The blue lines show the best-fitting linear relationships (Spearman correlations; *N*=25, ρ=0.32, *P*=0.12 for 2010; *N*=28, ρ=0.42, *P*=0.027 for 2015), and the grey shaded areas show the corresponding 95 % confidence intervals. Each point is labelled with the two letter code of the corresponding country, as defined by Eurostat (https://ec.europa.eu/eurostat). There is notable variation in reporting practices between countries; additionally, in 2010, most reporting laboratories used CLSI (Clinical and Laboratory Standards Institute) guidelines, whereas in 2015 most used EUCAST (European Committee on Antimicrobial Susceptibility Testing) guidelines. Generally, meningitis isolates should be consistently reported as PNSP if their MIC is above 0.06 μg ml^−1^, and non-meningitis isolates should be consistently reported as PNSP if their MIC is above 2 μg ml^−1^. Reporting is less consistent regarding whether non-meningitis isolates with an MIC above 0.06 μg ml^−1^, but equal to or below 2 μg ml^−1^, are defined as PNSP. National penicillin consumption was quantified as Defined Daily Doses (DDD). Data are from the ECDC (https://www.ecdc.europa.eu/).

*Streptococcus pneumoniae* (the pneumococcus) is a common Gram-positive commensal of the human, typically infant, nasopharynx. Outpatient consumption of antibiotics, particularly in children, is assumed to represent the main selection pressure driving the evolution of pneumococcal antibiotic resistance. Correspondingly, carriage surveys in infants often identify recent antibiotic consumption as a risk factor for carrying penicillin-non-susceptible pneumococci (PNSP) [1–5]. Transmission dynamic modelling suggests this is likely to reflect treatment clearing carriage of penicillin-susceptible pneumococci, reducing their ability to block the acquisition of PNSP through competition [6]. At both the regional [1] and continental [7] scales, this results in a linear relationship between outpatient use of penicillins and the proportion of *S. pneumoniae* isolates that are penicillin non-susceptible, which typically spans the range of ~1 % to over 40 % [7]. Analysis of recent data from the European Centre for Disease Prevention and Control (ECDC; Fig. 1) shows this relationship across European countries. This trend has persisted despite the introduction of pneumococcal polysaccharide conjugate vaccines (PCVs) [8], which protect against a subset of strains [9], although the correlation appears to have weakened substantially relative to the pre-PCV period [7].

Pneumococci themselves drive substantial paediatric antibiotic consumption. *S. pneumoniae* is one of the two primary aetiological agents of acute otitis media (AOM) [10], which affects 80 % of children before the age of 3 years, and correspondingly represents one of the main reasons for prescribing antibiotics to children [11, 12]. Reducing incidence of this usually non-critical *S. pneumoniae* disease, or changing treatment recommendations, should substantially reduce bystander selection on commensal bacteria to become more resistant to penicillin [13]. Yet efforts to reduce PNSP prevalence through lowering antibiotic consumption have had limited impact. In Iceland, reduced carriage of a multidrug-resistant *S. pneumoniae* strain was observed over a decade in which there was a one-third reduction in paediatric antimicrobial consumption, but there was no overall fall in PNSP carriage over this period [14]. Similarly, a 20 % reduction in outpatient antibiotic consumption over a decade in Sweden was contemporaneous with the curtailing of a multidrug-resistant outbreak, but did not result in a decrease in PNSP carriage [15]. A community intervention trial in the USA achieved a similar decrease in antibiotic consumption, but did not show a decrease in PNSP carriage in the 6 month post-intervention period relative to control regions [16]. Furthermore, there was no decline in the proportion of PNSP isolated from AOM cases over a 5 year period in Israel, despite an almost one-third decline in antibiotic prescribing to children under the age of 5 years, mainly driven by lowered penicillin dispensing [17]. Therefore, it seems PNSP persist among circulating strains at their established prevalences for years after changes in antibiotic consumption.

In 2013, the Centers for Disease Control and Prevention (CDC) estimated antibiotic-resistant *S. pneumoniae* were causing 1.2 million infections in the USA annually, resulting in 7000 deaths [18]. Mortality typically results from PNSP infections that are rarer and more invasive than AOM, such as pneumonia, bacteraemia and meningitis. These diseases have high case fatality rates even when pneumococci are antibiotic susceptible [19]; hence, meta-analyses have sought to identify the increase in mortality attributable to PNSP. Such a study of pneumococcal pneumonia found PNSP were associated with a relative risk of death of around 1.3 [20], although it can be difficult to adjust for the confounding associations between resistance and differences in pneumococcal pathogenicity [8]. A similar analysis of invasive pneumococcal disease across Europe found patients had a relative risk of death of 1.91 if the pathogen were a PNSP, although meningitis was the only clinical presentation that was individually significantly associated with worse outcomes when caused by PNSP [21]. Combining such information with continent-wide surveillance data estimated PNSP (including those resistant to additional antibiotics) caused over 5000 bloodstream infections across Europe annually, with an attributable mortality of 316 in 2015 [22].

## Population genomic analyses of penicillin-non-susceptibility

There is extensive variation in isolates’ levels of penicillin non-susceptibility, which reflects the complex genetics of *S. pneumoniae* resistance to β-lactams. For most clinical presentations, only minimum inhibitory concentrations (MICs) above 2 μg ml^−1^ cause difficulties for treatment, and this breakpoint was adopted in 2008 [23]. However, the poor penetration of penicillin through the blood–brain barrier [22] means isolates with an MIC above 0.06 μg ml^−1^ are classed as resistant in cases of meningitis.

The first reported PNSPs, exhibiting MICs up to 0.2 μg ml^−1^, were from Massachusetts (USA) in the 1960s [24]. Higher-level resistance was detected in Australia and Papua New Guinea later the same decade (maximum MIC of 2 μg ml^−1^), and in Minnesota (USA; maximum MIC of 4 μg ml^−1^) and South Africa (maximum MIC of 12 μg ml^−1^) in the 1970s [25]. Radiolabelling experiments demonstrated the non-susceptibility of these South African isolates resulted from alteration to as many as five of the six pneumococcal penicillin-binding proteins [26]. These changes enabled the proteins to maintain their physiological role in cell wall metabolism, while having lowered affinity for penicillin. Their coding sequences were found to have a mosaic structure, with short segments exhibiting high (>10 %) divergence relative to the orthologous sequences in penicillin-susceptible pneumococci [27, 28]. This was the consequence of recombination with closely related species that also inhabit the human oronasopharynx, such as *Streptococcus oralis* and *Streptococcus mitis* [28, 29]. The alterations conferring penicillin-non-susceptibility seem likely to have either arisen as neutral variation [30], or emerged under selection as resistance-conferring mutations [31], in these donor species prior to being imported into *S. pneumoniae* through transformation.

**Fig. 2.**
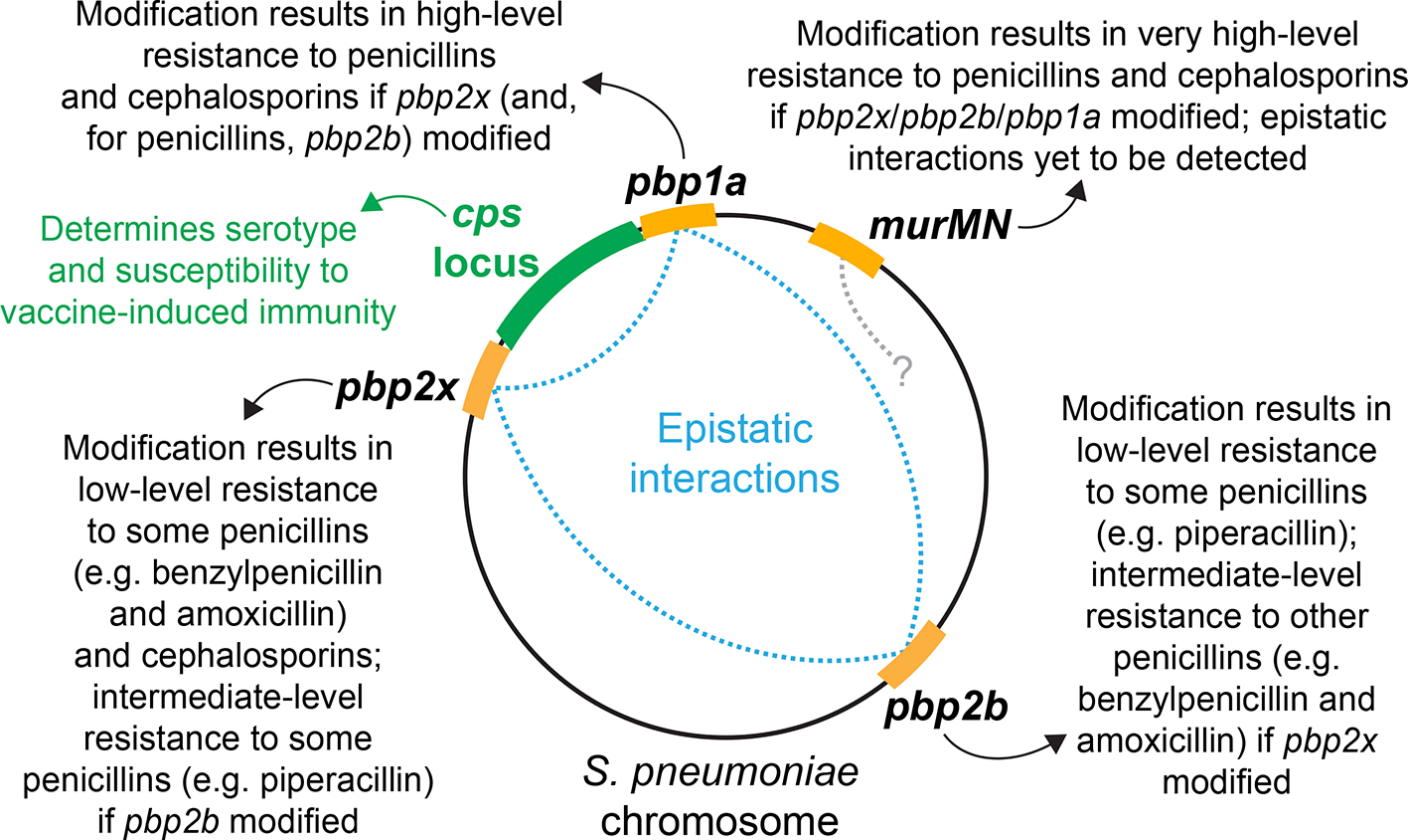
Summary of the genetic determinants of pneumococcal β-lactam-non-susceptibility, and their relative positioning in the bacterium’s chromosome.

Further molecular microbiology work elucidated the relationships between non-susceptibility phenotypes and particular penicillin-binding proteins (Fig. 2). Some penicillins (e.g. amoxicillin) and third-generation cephalosporins are highly effective at inhibiting Pbp2x and, therefore, changes to *pbp2x* alone confer low-level non-susceptibility [32, 33]. Although piperacillin (commonly prescribed in combination with tazobactam) is also most active against Pbp2x [34], alterations to *pbp2b* are the first step mutations for low-level non-susceptibility to this drug [35]. Alterations to both *pbp2x* and *pbp2b* are required for elevated MICs to all penicillins [32]. Additional changes to Pbp1a are necessary to confer high-level resistance to both penicillins and cephalosporins [32, 33]. Further increases in MICs to both classes of β-lactam can be achieved through modification of the *murMN* genes, which alters the structure of the cell wall [36, 37]. Only a few years after the first PNSP were identified, a wide variety of *pbp2x*, *pbp2b* and *pbp1a* alleles were observed throughout the species, with extensive exchange between genotypes through transformation [38].

Such recombination means there is limited linkage disequilibrium across genetically diverse *S. pneumoniae* populations, increasing the resolution of genome-wide association analyses in the species. Consequently, an analysis of β-lactam non-susceptibility across two independent population samples was able to confirm the dominant contribution of the three frequently-altered penicillin-binding protein genes (*﻿pbp2x*﻿*﻿/*﻿*2b* */* *1a*) with sufficient precision to indicate the most strongly associated gene segments [39]. These encoded the transpeptidase domain of Pbp2x, the transglycosylase domain of Pbp1a and the dimerization domain of Pbp2b. Loci within Pbp1a showed the strongest differential association with penicillin and cephalosporin resistance, suggesting different mechanisms of high-level resistance to these β-lactam classes. Few other loci were found to contribute to non-susceptibility in these populations, with almost all of the PNSP phenotypes explicable via SNPs co-detected as contributing to resistance in both populations. However, the low overlap of these SNPs with the set of polymorphisms consistently identified in highly resistant *S. pneumoniae* from the USA [40] gives an indication of the challenge of specifying the causative changes that underlie the PNSP phenotype (Table 1).

**Table 1.** Association of Pbp2x/2b/1a polymorphisms with penicillin-non-susceptibility Li and colleagues found 27 positions within the Pbp2x/2b/1a transpeptidase domains that were consistently altered in highly PNSP isolates from the USA (MIC of at least 4 μg ml^−1^) relative to penicillin-susceptible *S. pneumoniae* [40]. These have a relatively low overlap with the set of sites correlated with penicillin-non-susceptibility across two independent populations in a genome-wide association study [39]. This highlights the difficulty of identifying the causative changes underlying the penicillin-non-susceptibility phenotype in global collections of isolates.

Penicillin-binding protein	Amino acid position	Co-detected by GWAS
1a	T371	No
1a	E397	No
1a	N405	No
1a	T540	No
1a	N546	Yes
1a	A550	No
1a	T574	No
1a	S575	Yes
1a	Q576	Yes
1a	F577	Yes
1a	L583	Yes
1a	A585	Yes
2b	Q427	No
2b	T446	No
2b	E476	No
2b	T489	No
2x	R254	Yes
2x	M256	Yes
2x	T338	Yes
2x	I371	No
2x	G382	No
2x	R384	No
2x	T401	No
2x	N444	No
2x	S531	No
2x	L565	No
2x	S576	No

GWAS, genome-wide association study.

The *pbp2x*/*2b*/*1a* genes were also highlighted by phenotype-blind genome-wide epistasis analyses. Studies employing direct coupling analysis [41, 42] and pairwise mutual information [42, 43] methods identified the links between these genes as by far the strongest signals of co-evolution between spatially separate loci in the *S. pneumoniae* chromosome. This likely represents the fitness advantage of alterations at *pbp1a*, and often *pbp2b*, typically being contingent upon corresponding alterations at *pbp2x*. Alternatively, it may represent the importance of compensatory mutations, not directly involved in causing resistance, but instead maintaining the routine physiological functioning of the altered proteins [44]. Genes determining resistance to the components of co-trimoxazole were among the few other loci highlighted by both the association and epistasis analyses, likely representing co-selection for resistance to both classes of antibiotic [39, 41].

## Genomic epidemiology of penicillin-non-susceptible strains

**Fig. 3.**
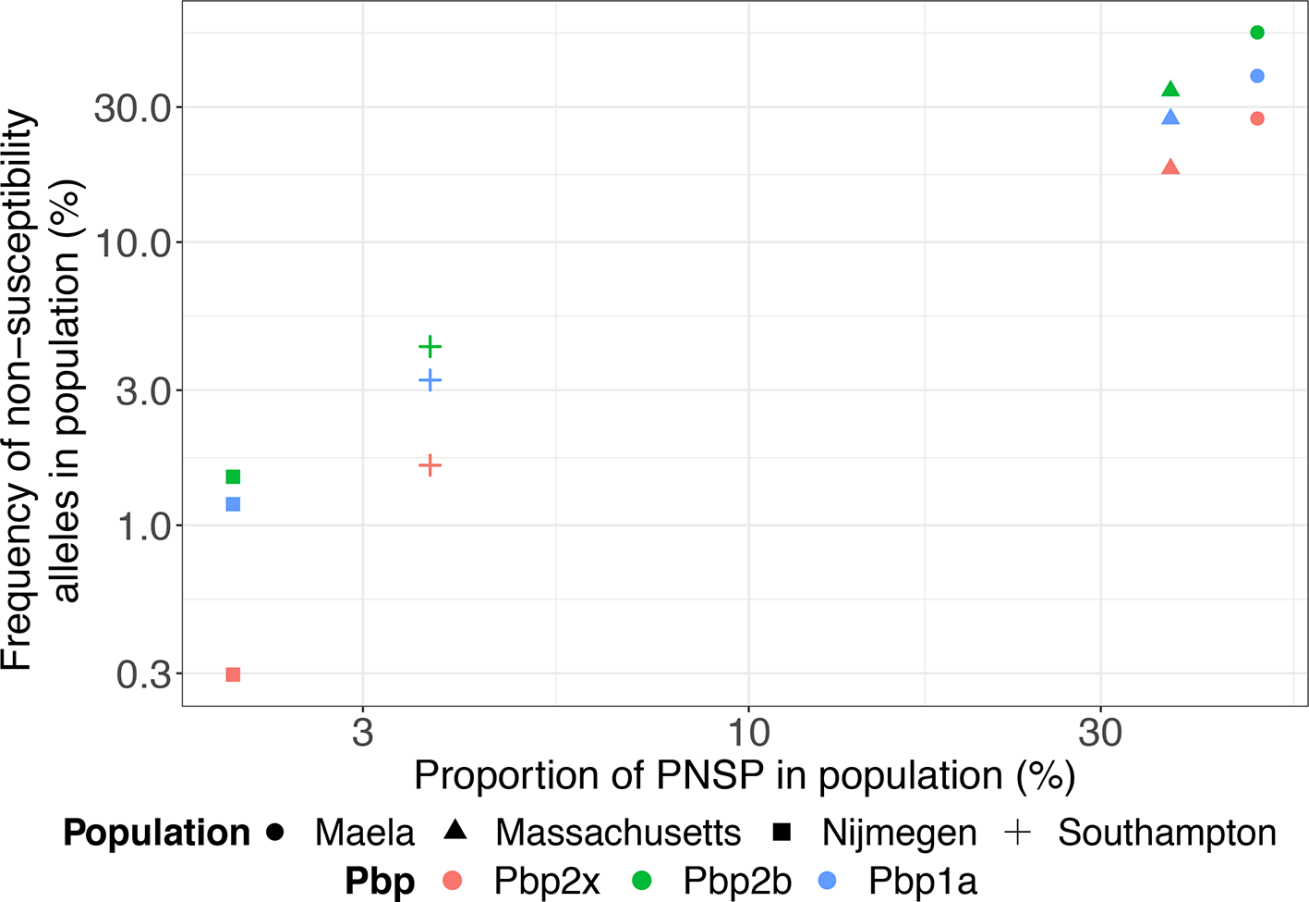
Distribution of penicillin-binding protein alleles associated with penicillin-non-susceptibility in four systematically sampled populations: carriage isolates from Maela (Thailand), Massachusetts (USA) and Southampton (UK), and disease isolates from Nijmegen (Netherlands). The sequence data are the set described by Corander and colleagues [74], in which alleles of penicillin-binding protein transpeptidase domains associated with non-susceptibility were identified through their deviation from a reference set of sequences from a susceptible isolate. The proportions of PNSP were reported for Maela and Massachusetts samples [39], assumed to be the same as for a similar carriage study from the UK for the Southampton samples [75], or estimated from national-level 2010 ECDC data (Fig. 1) for the Nijmegen samples.

The co-evolution of these resistance loci means *pbp2x*/*2b*/*1a* alleles associated with non-susceptibility accumulate in individual genotypes, and consequently these alleles’ frequencies are similar within a population, representing their underlying correlation with the prevalence of circulating PNSP (Fig. 3). The most successful PNSP clones, or strains [45], were originally defined by the Pneumococcal Molecular Epidemiology Network (PMEN) [46], which identified over 20 strains with penicillin MICs above 0.06 μg ml^−1^ by 2006. Many of these PNSP PMEN clones were associated with resistance to other classes of antibiotics, and were the vector by which the loci causing multidrug-resistance in pneumococci spread globally [47]. The evolutionary epidemiology of these strains has recently been described in detail using an international collection of pneumococcal genomic data [48, 49].

Phylodynamic analyses of PNSP PMEN strains confirms that they have rapidly transmitted worldwide [50], with strains common in a particular region typically having been imported multiple times, even within isolated locations [51, 52]. Reconstructing these genotypes’ evolutionary history reveals they appear to have acquired non-susceptibility alleles of the *pbp2x*/*2b*/*1a* genes over a short timeframe, and possibly simultaneously [38, 53]. Such emergence of the PNSP phenotype may occur sporadically within an otherwise susceptible lineage [45], or occur multiple times in parallel in a closely related set of isolates [51, 52], else a single acquisition of resistance may give rise to a widely disseminated lineage [50]. As yet, there is little evidence of intermediate genotypes that would suggest stepwise emergence of PNSP. This apparent saltational evolution could represent limited sampling of historical events, or the heterogeneous nature of *S. pneumoniae* transformation, which typically only imports a short segment of sequence from a divergent strain once every few years, but intermittently facilitates multiple large recombinations [54].

PNSP evolution during their global spread typically involves an accumulation of variation through further recombination, which is generally concentrated at ‘hotspots’ of transformation events [50]. The *pbp2x*, *pbp2b* and *pbp1a* genes are frequently modified by such sequence exchange [45, 51, 52, 55], suggesting they may be adapting to changing β-lactam prescribing practices. Additionally, *pbp2x* and *pbp1a* closely flank the capsule polysaccharide synthesis (*cps*) locus (Fig. 2), which determines the pneumococcal serotype and, therefore, whether the bacterium is targeted by immunity induced by the PCVs [8]. ‘Serotype switching’ recombinations that enable vaccine escape can, therefore, also affect *pbp2x* and *pbp1a*. Laboratory experiments have demonstrated a single large transformation-mediated homologous recombination can both decrease susceptibility to penicillin and change an isolate’s serotype [56]. Conversely, serotype switches in a PNSP strain can cause *pbp2x* and *pbp1a* to revert to susceptibility-associated alleles [51].

## Genomic surveillance in the post-vaccine era

*S. pneumoniae* surveillance is critical for understanding the impact of PCVs. As these vaccines targeted many serotypes expressed by PNSP strains, it was hoped they would cause a sustained reduction in *S. pneumoniae* antibiotic resistance [57]. However, following the first introduction of the 7-valent PCV in the USA, the inital observed reduction in the fraction of invasive pneumococcal disease caused by PNSP proved only temporary, and ‘bounced back’ to its original proportion a few years later [58]. This mirrored the typically stable frequency of PNSP in *S. pneumoniae* carriage populations [59, 60]. In the USA, these changes largely reflected the elimination of multiple vaccine-targeted lineages with high penicillin MICs, and the contemporaneous emergence of PNSP not targeted by the 7-valent PCV [61]. These PNSP increasing post-PCV comprised a few highly-resistant strains, typically of serotype 19A, as well as diverse PNSP with lower penicillin MICs [61, 62]. Consequently, the updated 13-valent PCV now includes the 19A capsule as an antigen [8], although vaccine-induced immunity has yet to completely eliminate this serotype [63, 64]. Worldwide genomic epidemiology has, nevertheless, identified a general decrease in the prevalence of PNSP in the initial years following the introduction of the 13-valent PCV, albeit with multiple PNSP strains not targeted by the vaccine increasing in frequency [65]. These include a lineage of serotype 11A in Spain [66], multiple genotypes expressing serotype 35B in the USA [67], and isolates of serotype 24F emerging as a major cause of infant meningitis in France [68].

Some countries are now switching to routine whole-genome sequencing for the surveillance of PCV impact on pneumococcal disease. This can be a cost-effective approach, as both population structure and serotypes can be efficiently inferred from these genetic data [48, 49, 69, 70], in addition to most antibiotic-resistance phenotypes [71]. However, the diversity of *pbp2x*/*2b*/*1a* alleles makes predicting penicillin-non-susceptibility from the genome challenging, particularly considering the importance of accurately ascertaining the MICs for multiple classes of β-lactams for informing treatment strategies [23]. Two approaches have proved effective at addressing this challenge using a database of Pbp2x/2b/1a transpeptidase domain sequences and MICs maintained by the CDC [71, 72]. The ‘mode MIC’ method uses a typing scheme created from these domain sequences, and predicts new isolates to have the MIC most commonly observed in the set of isolates in the database with the most similar Pbp2x/2b/1a profile [40]. The ‘random forest’ approach employs a machine learning method trained on existing data to predict the MICs directly from the transpeptidase domain amino acid sequences [40, 71]. Both methods have demonstrated very high (>98 %) correspondence with microbiological susceptibility typing when providing information relevant for treatment, with retesting of discrepant examples often concurring with the genomic prediction [40, 71]. While both are most effective when applied to already-characterised proteins, the random forest method is the more effective technique when applied to previously unobserved Pbp2x/2b/1a sequences [40, 73]. Consequently, genomics promises to play an increasingly prominent role in the evaluation of new vaccines and treatment practices that may be employed to reduce PNSP disease.

## Data bibliography

1. ECDC. *Antimicrobial Resistance Surveillance in Europe 2010. Annual Report of the European Antimicrobial Resistance Surveillance Network (EARS-Net)*. Stockholm: European Centre for Disease Prevention and Control (2011).

2. ECDC. *Antimicrobial Resistance Surveillance in Europe 2015. Annual Report of the European Antimicrobial Resistance Surveillance Network (EARS-Net)*. Stockholm: European Centre for Disease Prevention and Control (2017).

3. ECDC. *Surveillance of Antimicrobial Consumption in Europe, 2010*. Stockholm: European Centre for Disease Prevention and Control (2013).

4. ECDC. A*ntimicrobial Consumption 2015. Annual Epidemiological Report for 2015*. Stockholm: European Centre for Disease Prevention and Control (2018).
